# Expression of Concern: Multiobjective intuitionistic fuzzy programming under pessimistic and optimistic applications in multivariate stratified sample allocation problems

**DOI:** 10.1371/journal.pone.0353916

**Published:** 2026-07-16

**Authors:** The PLOS One Editors

The *PLOS One* Editors issue this Expression of Concern because this article [1] was identified as one of a series of submissions for which we have concerns about the peer review process. Readers are advised to interpret the article [1] with caution.
