# Supplementary material for: Multiobjective intuitionistic fuzzy programming under pessimistic and optimistic applications in multivariate stratified sample allocation problems
Source: PLoS One. 2023 Apr 21;18(4):e0284784. doi: 10.1371/journal.pone.0284784 (PMC10121053; doi:10.1371/journal.pone.0284784)
Supplement: Attachment — Submitted filename: Response to Reviewers.docx [file pone.0284784.s001.docx]

**Reviewer's Responses to Questions**

Dear Editor

We thank you for your time and efforts on our manuscript.

Further, we take this opportunity to thank the anonymous reviewers for their valuable suggestions. The suggested major revisions have been efficiently carried out in the revised version of the manuscript.

All the changes have been highlighted in yellow.

Thanking you

On behalf of all authors

**Dr. Ahteshamul Haq**

**Reviewer #1:** The authors could provide good work. However, there are some concerns to be resolved.

1. The abstract is loosely written. It is not as informative as expected. A standard abstract must present, without leaving any doubt, the objective of the paper precisely; source of data (which is not present in your abstract) and analytical approach used; key findings and any policy implication and recommendations

**Response 1:** We updated the Abstract section of the manuscript on the reviewer's recommendation and the same is highlighted in the yellow.

2. The structure of the introduction is not in a standard form. first, we need to find the basics of your problems and then the main aspects and issues related to them. Do not pay attention to lots of details (especially the foundation of the problem).

**Response 2:** We updated the Introduction section of the manuscript, which is highlighted in yellow.

3. English presentation of the paper should be modified so as to be more readable.

**Response 3:** All the errors have been corrected in the revised manuscript.

4. I suggest the authors read and consider the studies performed by Alireza Goli et al. and their groups on the application of metaheuristic algorithms and supply chain optimization.

**Response 4:** The manuscript with the suggested author group’s references has been revised and updated.

5. Authors should improve the material selection criteria and methods section considering the latest published work in reputed journal.

**Response 5:** The manuscript has been revised and improved the criteria and method section.

6. The implications of the results should be discussed in more detail. The authors should provide managerial insights based on the output.

**Response 6:** The manuscript has been revised, and the updated with reviewer's recommendation and highlighted it.

**Reviewer #2:** The paper deals with the challenge of allocating resources for a multivariate stratified sample survey. The goal is to estimate population means for a fixed cost and this problem is formulated as a mathematical programming problem. The paper presents solution procedures using fuzzy programming optimistic and pessimistic methods.

The paper relevance and subject is interesting. However, it can be improved further.

1. The writing of the paper needs to be reconsidered. In many cases the sentences are either grammatically incorrect or simply incomplete. For instance, in the abstract L3, the word “respectively” is used incorrectly. Or in the second sentence of the introduction, I guess “When” needs to be replace with “while” and also the last part of the sentence “helps to keep the variance of the stratified sample” is incomplete.

**Response 2:** We updated the Abstract and Introduction section for the grammatical errors in the manuscript and highlighted it.

2. What is “s” in the notation

**Response 2:** S^2^ is population mean square in the notation and defined it in the revised manuscript.

3. I suggest make this sentence as some bullet points, providing definitions for each symbol: “The hth stratum, and denote stratum size, the population mean, weight, and population mean square for the hth ( ) stratum”.

**Response 2:** We denote the stratum size, population mean, weight, and population mean square for the h^th^ stratum in the revised and highlighted it.

4. Although the definitions and formulations in the introductions are known, proper references to the original studies should be provided.

**Response 2:** We updated the introduction in the revised manuscript as per the reviewer's recommendation and highlighted it.

5. The literature review provide no story and background to the current work. What is it that the authors find defective and how the use of fuzzy programming will address the shortcoming in the previous studies? I suggest reorganizing the background review in a way that the reader in informed about the gap and then authors need to clearly justify their proposal and how it will add value to the field before proceeding to methodology.

**Response 5:** Thanks for the suggestions in the revised paper; we have done as you recommended. Now the abstract as well as the conclusion section, we also added the application of the study. Moreover, the study is a multiobjective optimization problem that can not be solved directly by any optimization techniques. This paper uses optimistic and pessimistic methods with fuzzy programming for solving the multiobjective sampling problem. The method used is flexible and provides a better compromise solution for the problem. Several works on sampling MOOP were published by one of our co-authors and anyone can see the reference list.

6. As mentioned earlier, the methodology needs to be rewritten in a clear way. From the first sentence “The multiobjective multivariate stratified sampling (MOMSS) model where the variances form the objectives and cost work as a constraint.” Which I find difficult to grasp what the authors are trying to deliver. Later authors define aspiration level, reservation levels … which are not used in the following models. It is not also clear how the preference information is elicited from the DM? a priori? Is it possible for the DM to provide such information before having any options or knowledge on the possibilities?

**Response 6:** In this paper, we used optimistic and pessimistic methods with fuzzy programming to solve the multiobjective sampling problem. The method used is flexible and provides a better compromise solution for the problem. One of our co-authors published several works on sampling MOOP; anyone can see the reference list. In this paper, since we don’t know the goal for the several objectives, if we don’t, then we solve the problem as a single objective and determine the goal/aspiration level. It is standard practice for solving the MOOP without already defined goal values. However, we have updated the paper as per your suggestions.

We express our gratitude and sincere thanks to the anonymous reviewer for reading the manuscript and pointing out constructive and valuable comments to improve the quality of our paper.
